# Sensory evidence for complex communication and advanced sociality in early ants

**DOI:** 10.1126/sciadv.adp3623

**Published:** 2024-06-14

**Authors:** Ryo Taniguchi, David A. Grimaldi, Hidehiro Watanabe, Yasuhiro Iba

**Affiliations:** ^1^ Department of Natural History Sciences, Graduate School of Science, Hokkaido University, Sapporo, Hokkaido 060-0810, Japan.; ^2^ Division of Invertebrate Zoology, American Museum of Natural History, New York, NY 10024-5192, USA.; ^3^ Department of Earth System Science, Fukuoka University, Fukuoka, Fukuoka 814-0180 Japan.; ^4^ Department of Earth and Planetary Sciences, Hokkaido University, Sapporo, Hokkaido 060-0810, Japan.

## Abstract

Advanced social behavior, or eusociality, has been evolutionarily profound, allowing colonies of ants, termites, social wasps, and bees to dominate competitively over solitary species throughout the Cenozoic. Advanced sociality requires not just nestmate cooperation and specialization but refined coordination and communication. Here, we provide independent evidence that 100-million-year-old Cretaceous ants in amber were social, based on chemosensory adaptations. Previous studies inferred fossil ant sociality from individual ants preserved adjacent to others. We analyzed several fossil ants for their antennal sensilla, using original rotation imaging of amber microinclusions, and found an array of antennal sensilla, specifically for alarm pheromone detection and nestmate recognition, sharing distinctive features with extant ants. Although Cretaceous ants were stem groups, the fossilized sensilla confirm hypotheses of their complex sociality.

## Teaser

Primitive ants 100 million years ago had communicated with their nestmates via pheromones and lived in advanced societies.

## INTRODUCTION

Sociality is the most complex and advanced biological form of cooperation because it requires communication with other individuals. The evolution of sociality brought altruism to a selfish world, and, thus, it has been a key innovation in the history of life, spawning the large discipline of sociobiology (*1*–*3*). Among many social organisms, which are largely arthropods, the importance of ants (Hymenoptera: Formicidae) is reflected in their abundance as well as species and ecological diversity (e.g., herbivores, predators, and fungivores, with diverse symbionts). It is estimated that the world’s ant population is 20 quadrillion (20 × 10^15^) individuals, a biomass comparable to that of humans globally (*4*, *5*). Such pervasiveness makes ants an essential group in terrestrial ecosystems (*4*–*6*), all supported by sophisticated social systems such as complex communication and a division of labor with sterile workers (*7*, *8*), which have been investigated with behavioral experiments, genomics, physiology, and phylogenetic analyses (*8*–*12*). Ants, all living species of which are eusocial, probably split from their solitary wasp ancestors in the Early Cretaceous (*13*–*15*). The oldest definitive records can be traced to amber from the mid-Cretaceous of France and Myanmar [~105 to 100 million years (Ma) ago] (*16*, *17*). Their sociality has been inferred on the basis of adjacent preserved conspecifics (*17*–*19*), as well as the possession of a metapleural gland (*8*), making it uncertain if or how much they interacted cooperatively. Most recently, an adult and a pupal ant were found in the same piece of Myanmar amber and described as the same species (*19*), the authors interpreting this as evidence of eusocial brood care.

Rather than relying on statistical encounters of inclusions in amber pieces, we focused on a comparative approach of features intrinsic to the social communication systems of ants. Ants communicate with nestmates by various pheromones, such as for alarm, defense, recruitment, and trails (*20*). Unfortunately, preservation of pheromones in amber is extremely unlikely because they are low–molecular weight volatiles (e.g., terpenoid aldehydes, alcohols, alkanes, and ketones), and the amber itself is a terpene polymer (*20*–*22*). An ant fossil in Myanmar amber has been found to preserve internal organs putatively for social communication, but functions are ambiguous of the partially degraded remains of pheromonal glands and gross brain structure (*23*). An alternative approach involves the sensory organs that receive the communication signals. Ant pheromones are detected by antennal sensilla, which are micrometer-scale sensory cuticle hairs with neuronal innervation (*9*, *24*–*26*). Each sensillum type is morphologically and functionally distinctive (*27*, *28*). Sensory organs on the exoskeleton of insects, although microscopic, are externally chitinous and thus can be well fossilized in amber, possibly the only direct evidence for ant social communication. However, the resolution of traditional visualization [e.g., photomicrography and x-ray computed tomography (CT) (0.5-μm resolution now, although not necessarily in amber)] is insufficient for these minute sensory organs because of light scattering and low-density differences. For this reason, previous studies have not examined the sensilla of fossilized ants (*17*–*19*).

Here, we analyzed the antennae of one of the earliest ants, *Gerontoformica gracilis* (Barden and Grimaldi, 2014) (*29*), preserved in 100-million-year-old Cretaceous amber from Kachin State in northern Myanmar with a partially destructive method. This involved developing an original rotating visualization technique for amber to perform high-resolution imaging from multiple angles via confocal laser scanning microscopy (CLSM), reducing the optical aberrations. This method allowed us to visualize the microstructure and distribution of the antennal sensilla, thus reconstructing the communication systems.

## RESULTS

**Fig. 1.**
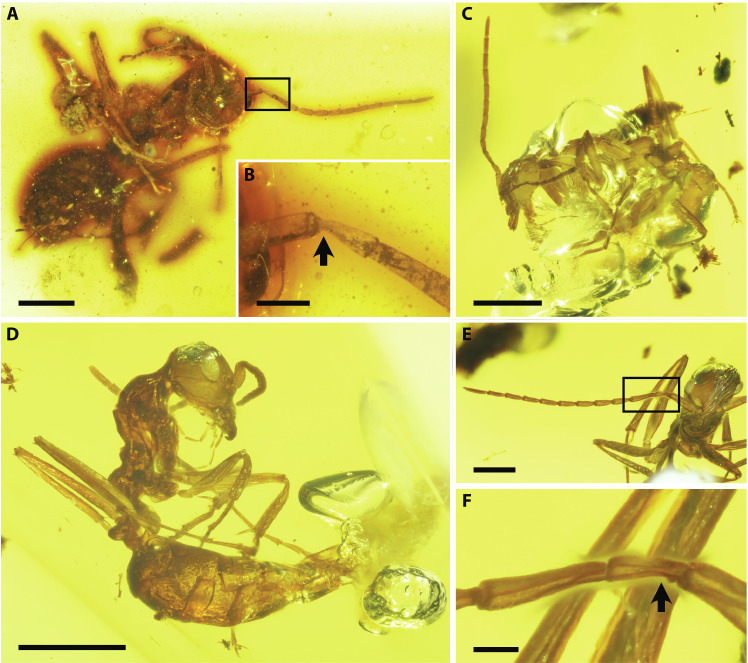
Photographs of fossilized ants, *G. gracilis*. (**A**) AMNH JZC Bu109, ventral view. (**B**) Enlarged image of the framed area in (A). (**C**) Specimen A in AMNH Bu-KL B1-21, lateral view. (**D**) Specimen B in AMNH Bu-KL B1-21, right lateral view. (**E**) The head of specimen B, frontal view. (**F**) Enlarged image of the framed area in (E). Specimen B shows the well-preserved forward-projecting right antenna (E). Pedicel constrictions indicating the inner lateral sides of antennae are marked by arrows in (B) and (F). Scale bars, 1 mm [(A), (C), and (D)], 0.2 mm (B), 0.5 mm (E), and 0.1 mm (F).

Macroscale characters of the antennae (>50 μm) were examined using macrophotography of three fossilized female *G. gracilis* ants preserved in Cretaceous amber [one ant in amber pieces AMNH JZC Bu109 and two ants (specimens A and B) in AMNH Bu-KL B1-21] (Fig. 1 and fig. S1). The specimens, selected from a series of conspecific specimens, had well-preserved antennae projected away from the body (Fig. 1). Each antenna comprises 12 segments (a scape, a pedicel, and a flagellum with 10 flagellomeres), as in extant adult females (cf. 13 segments with 11 flagellomeres in males); it is geniculate between the scape and pedicel. The pedicel is constricted on one side as in extant ants (Fig. 1, B and F) (*27*), allowing a landmark to the antennal inner lateral surface. Here, only the right antenna of specimen B in AMNH Bu-KL B1-21 could be accurately defined for the dorsal, ventral, inner, and outer surfaces because it was preserved linearly in the same plane (Fig. 1E). This specimen, therefore, was used for mapping the sensilla distribution patterns on the antenna.

**Fig. 2.**
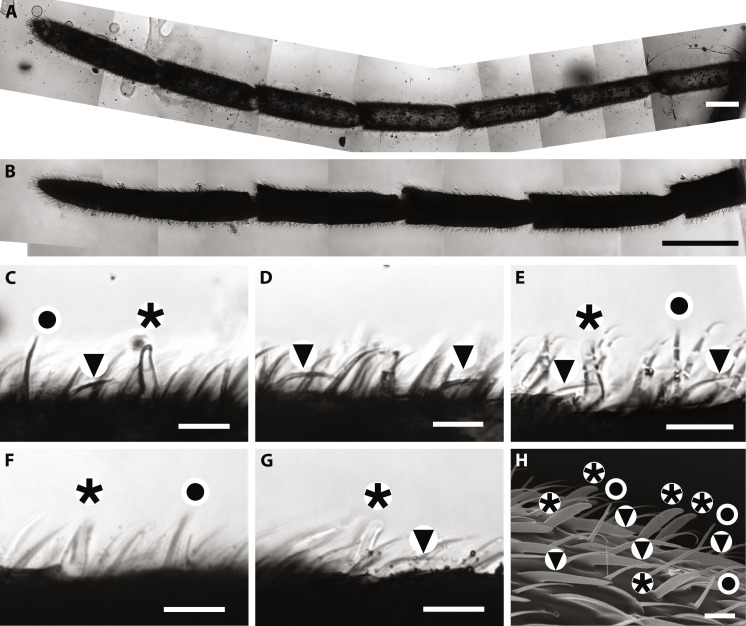
Antennae and their sensilla of fossilized *G. gracilis* imaged via CLSM and via SEM in extant *F. sanguinea*. (**A**) Left antenna of AMNH JZC Bu109. (**B**) Right antenna of specimen A in AMNH Bu-KL B1-21. (**C** and **D**) Antennal sensilla of AMNH JZC Bu109. (**E**) Antennal sensilla of specimen A in AMNH Bu-KL B1-21. (**F** and **G**) Antennal sensilla of specimen B in AMNH Bu-KL B1-21. (**H**) Antennal sensilla of *F. sanguinea*. Sensilla basiconica are marked by asterisks, trichodea curvata by triangles, trichodea by circles, and other hairlike sensilla without any marks are chatica or trichoid-II in (C) to (H). Scale bars, 100 μm [(A) and (B)] and 10 μm [(C) to (H)].

**Fig. 3.**
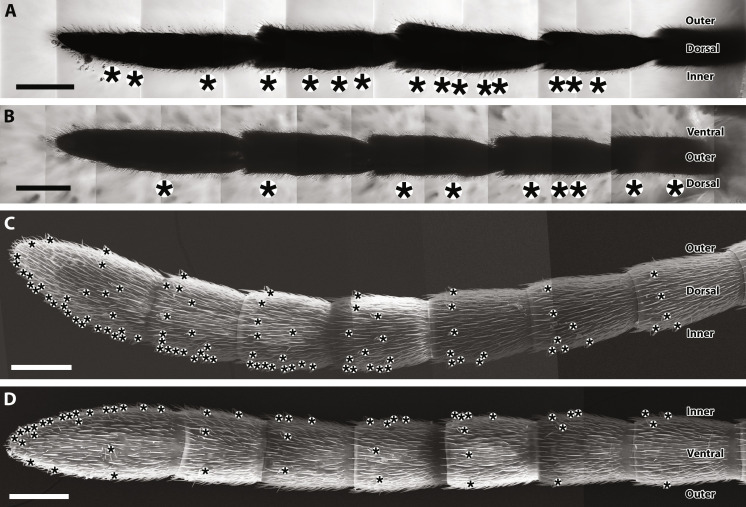
Distribution pattern of right antennal sensilla basiconica in fossilized *G. gracilis* and extant *C. quadrinotatus*. (**A** and **B**) *G. gracilis*. (**C** and **D**) *C. quadrinotatus*. Sensilla basiconica marked by asterisks are biased on the dorsal and inner surfaces on the antennae. Scale bars, 100 μm.

The microsensilla of all three fossilized antennae (the left antenna from AMNH JZC Bu109 and each right antenna of specimens A and B from AMNH Bu-KL B1-21) were visualized via the thin-sectioning and CLSM technique developed by Taniguchi *et al.* (*30*) (Figs. 2, A to G, and 3, A and B). All sensilla were classified into four morphotypes and identified as distinct sensillum types in extant ant taxa (*27*, *31*, *32*). Previous studies used different terminology for sensillum types, so we followed Ramirez-Esquivel *et al.* (*28*) because they compiled terminology from many previous studies. Sensilla basiconica are thumb-shaped pegs; sensilla trichodea are minute hairs tilted considerably toward the proximal direction of the antenna compared to the other sensilla (Fig. 2, C and E to H). Sensilla trichodea curvata are curved, sword-like pegs (Fig. 2, C to E, G, and H). The most abundant slender hairs are thought to be either sensilla chaetica or trichoid II (Fig. 2, C to H). In extant material, these two sensillum types are distinguished only based on ultrastructure observed with electron microscopy. These observations are too difficult for fossils in amber because the inclusions cannot be exposed and surface-scanned due to their fragility. Such diagnostic ultrastructure cannot be visualized even via our method; thus, we have left the possibility of two types here.

Because laser transmission does not penetrate the fossilized antennal cuticle (Fig. 2, A and B), to visualize and map sensillum distributions, after one surface was analyzed via CLSM, the thinned amber piece, including the antenna, was rotated 90° and ground/polished again, so that we could visualize the orthogonal surface (fig. S2). This two-time CLSM analysis of the specimen allowed observation throughout the dorsal, ventral, and inner and outer surfaces of the fossilized antenna. It revealed a concentrated pattern in the distribution of sensilla basiconica on the dorsal and inner surfaces (Fig. 3, A and B). The same dorsal-inner distribution of sensilla basiconica was also observed in all six extant species via scanning electron microscopy (SEM) (Fig. 3, C and D, and fig. S3).

## DISCUSSION

On extant ant antennae, there are eight types of sensilla based on their structure (*28*). These include five types of elongated protrusions (sensilla basiconica, trichodea, trichodea curvata, chaetica, and trichoid-II) and three types of peg-in-pit sensilla (sensilla ampullacea, coeloconica, and coelocapitular). The present study revealed at least four morphotypes of protruding sensilla on the Cretaceous *Gerontoformica* ants, all found in extant ants: sensilla basiconica, trichodea, trichodea curvata, and chaetica/trichoid-II (Fig. 2, C to H).

Sensilla trichodea curvata are found uniquely in ants and occur in all ant taxa. Previous studies found that this sensillum type occurs in more than 60 species in 10 subfamilies of extant Formicidae (*25*, *28*, *31*–*35*). Sensilla trichodea curvata are considered homologous to plate-like sensilla placodea of some other hymenopterans (*33*). The transformation from flat plates embedded on the antennal surface (sensilla placodea) to protruding pegs (sensilla trichodea curvata) allows ants to detect molecules more effectively on a larger surface area. In extant ants, electrophysiological analysis has shown that sensilla trichodea curvata detect alarm pheromones (*24*, *27*, *28*). In detecting alarm pheromones, ants display aggressive or escape behavior in response to intruders (*36*, *37*). †*Gerontoformica* already acquired this unique ant sensillum by 100 Ma ago (Fig. 2, C to E and G) and correspondingly is likely to have used chemical alarm signaling. Acquiring alarm communication must have allowed stem ants to coordinate and mobilize nestmates to great advantage in inter- and intraspecific competition, attempted predation, and the invasion of territorial space, just like extant social insects (*2*, *38*).

Sensilla basiconica are contact chemoreceptors for substrate molecules and their distribution patterns on hymenopteran antennae are correlated with ecology. For instance, sensilla basiconica concentrated on the ventral surface in some parasitic wasps optimizes them for detecting molecular traces of their hosts (*39*). In eusocial hymenopterans, such as ants, hornets (Vespidae), and honey bees (*Apis*), the sensilla basiconica are usually distributed from the dorsal to the inner surface (Fig. 3, C and D, and fig. S3) (*27*, *28*, *40*, *41*). The sensilla basiconica of eusocial species assist in distinguishing nestmates from intruders based on colony-specific cuticular hydrocarbon blends on the body surface (*9*, *42*). The concentrations of these antennal sensilla on the dorsal and inner surfaces optimize nestmate recognition during antennation with other ants (*27*, *32*). The same concentration of sensilla basiconica was observed in *Gerontoformica* as in modern ants (Fig. 3, A and B). They likely identified their nestmates through chemosensory contact using their antennae like extant ants.

The presence on the antennae of *Gerontoformica* of abundant sensilla trichodea curvata and the concentration of sensilla basiconica on dorsal and inner surfaces indicate that these early, stem-group ants were adapted for social chemical communication via pheromones, as in modern ants. As such, this provides compelling, independent support for hypotheses that ants from the mid-Cretaceous were eusocial.

This raises the question posed by Grimaldi and Agosti (*43*): If early ants were eusocial, and eusociality confers substantial ecological advantages, why were ants so rare for the entire first half of their known existence? Throughout the Cretaceous, ants comprised 1% or less of individual insects in fossil deposits (the primary source of ant fossils is amber, and they are scarce in sedimentary rocks) (*43*, *44*). It was not until the late Paleocene and Early Eocene that their abundance climbed to 10%, and not until the Neogene that it exceeded 20%. It could be argued that ants in Myanmar amber were not rare because hundreds of specimens and dozens of species are known (*17*). However, these have been found in huge, commercial-scale excavations of hundreds of tons of amber; all other Cretaceous deposits are a small fraction of this. In addition, noncommercial, unbiased samples of Myanmar amber show that ants comprise 1% of the inclusions (*45*).

The only explanation for this seems to be the colony size. Early ants probably lived in small colonies of several dozens, similar to modern amblyoponines and other basal ants today, and it was not until the origins of ant groups that formed immense colonies of many thousands in the Cenozoic that they became an overwhelming ecological force. Sophisticated social communication seems to have well preceded the ecological rise of ants.

## MATERIALS AND METHODS

### Provenance

Ant specimens in Burmese amber were acquired legally by the AMNH between 1999 and 2010 through purchase from state-licensed dealers in Myitkyina, Kachin state, northern Myanmar (*45*, *46*). The age of Kachin amber has been dated as ~100 Ma old, near the boundary between the Early and Late Cretaceous, based on U-Pb dating of zircons and biostratigraphic analysis (*47*, *48*). At the time of acquisition, Kachin amber was the only commercially available source of Burmese amber, before the discovery, ~2016, of commercial-scale amber from Tilin, central Myanmar, which is Campanian age, ~72 Ma (*49*).

### Fossil materials

The two focal amber pieces derive from Kachin State in northern Myanmar. Both pieces are deposited in the American Museum of Natural History with the collection numbers AMNH JZC Bu109 and AMNH Bu-KL B1-21, selected for partial destructive analysis from among a large series of specimens for the species. JZC Bu109 contains one fossilized ant, and Bu-KL B1-21 contains two fossilized ants (specimens A and B) (Fig. 1). The lack of wings and the presence of abdominal stings indicate that they are adult females. These fossils were identified as *G. gracilis* (Barden and Grimaldi, 2014) based on the taxonomic systematics in a previous study (*19*, *50*) (genus diagnosis: bidentate mandible fitting against clypeus at rest, and clypeus with setae and without lobate process; species diagnosis: mesonotum without transverse ridge, abdominal segment IV without constriction, petiole longer than tall, from Kachin amber, mesosoma lumpy, and mesometanotal and metanotopropodeal sulci anteroposteriorly broad) (fig. S1). The age of Kachin amber is almost as old as the earliest fossil records of ants, and *Gerontoformica* is the oldest ant genus (*16*–*18*). These specimens are, therefore, ideal for reconstructing the communication systems of ants in their early evolutionary stages.

### Methods

Macrophotos of the fossils were taken using a Canon EOS 5DS digital camera with a Canon MP-E 65 mm macro lens (F2.8, 1× to 5×) and a ZEISS LED Cold Light Source Dual Pipe Light System. The amber pieces were immersed in clove oil (Wako Pure Chemical Industries) to cover the surface imperfections and improve optical resolution. We used Helicon Focus 8.1.2 (Helicon Soft) to focus-stack the images that we obtained. The specimens were scanned via a microfocus x-ray CT system (60 kV; Bruker SKYSCAN 2214) at Tomakomai Industrial Technology Center (Hokkaido, Japan). The voxel sizes are 4.2 μm^3^ in JZC Bu109 and 3.5 and 3.0 μm^3^ in specimens A and B in Bu-KL B1-21, respectively. The CT data were rendered with Amira 3D 2022.2 (Thermo Fisher Scientific).

We applied the amber thin-sectioning technique developed by Taniguchi *et al.* (*30*) to visualize antennal microsensilla and their distribution pattern. One antenna of each ant fossil was cut off with its amber matrix using a printed circuit board cutter (HOZAN TOOL INDUSTRIAL Co. Ltd.) and a diamond wire saw (Pepaless Co. Ltd.); the cut pieces were then ground and polished to ~100-μm thickness using a diamond cup wheel (NIPPON DIAMOND Co. Ltd.). The antenna specimens in thinned amber pieces were visualized with a Nikon A1-Rsi CLSM system in the Nikon Imaging Center at Hokkaido University (using a Nikon Apo LWD 40× WI λS DIC N2 lens with 636.1-nm laser for JZC Bu109 and a Nikon Plan Apo VC 60× WI DIC N2 lens with 487.4-nm laser for Bu-KL B1-21). After being observed and imaged in one position, the specimens were rotated 90° and ground/polished again to be observed and imaged from different angles (fig. S2). This original rotation imaging of amber inclusions enabled us to visualize the entire distribution of micro sensilla on the antennal surface. A second view was also prepared with the A1-Rsi CLSM system. Images were stacked with Helicon Focus 8.2.3.

To compare the sensillum distribution patterns with fossils, we also observed antennae of six extant ant species belonging to four subfamilies: *Camponotus quadrinotatus* Forel, 1886; *Formica sanguinea* Latreille, 1798 (Formicinae); *Aphaenogaster famelica* (Smith, 1874); *Tetramorium tsushimae* Emery, 1925 (Myrmicinae); *Dolichoderus sibiricus* Emery, 1889 (Dolichoderinae); and *Pachycondyla chinensis* (Emery, 1895) (Ponerinae). These ants were ultrasonically cleaned in 50% acetone, fixed in 4% OsO_4_ in 50% acetone, dehydrated gradually by changing acetone concentrations, and air-dried. We then cut off their heads with antennae and coated them with platinum-palladium using a Hitachi E-1030 ion sputter. The prepared antennae were observed via a Hitachi S-4800 SEM system (magnification, ×180 to ×2000). All figures are adjusted and compiled via Serif Affinity Designer 1.10.8.

Specimens were ethically acquired from state-licensed dealers between 1999 and 2010 (also see the “Provenance” section).
