# Supplementary material for: Sensory evidence for complex communication and advanced sociality in early ants
Source: Sci Adv. 2024 Jun 14;10(24):eadp3623. doi: 10.1126/sciadv.adp3623 (PMC11177930; doi:10.1126/sciadv.adp3623)
Supplement: Supplementary file 1 — Figs. S1 to S3 [file sciadv.adp3623_sm.pdf]

Science Advances
AAAS

Supplementary Materials for

Sensory evidence for complex communication and advanced sociality in early ants

Ryo Taniguchi *et al.*

Corresponding author: Ryo Taniguchi, ryoxtaniguchi@eis.hokudai.ac.jp

Sci. Adv. **10**, eadp3623 (2024)
DOI: 10.1126/sciadv.adp3623

This PDF file includes:

Figs. S1 to S3

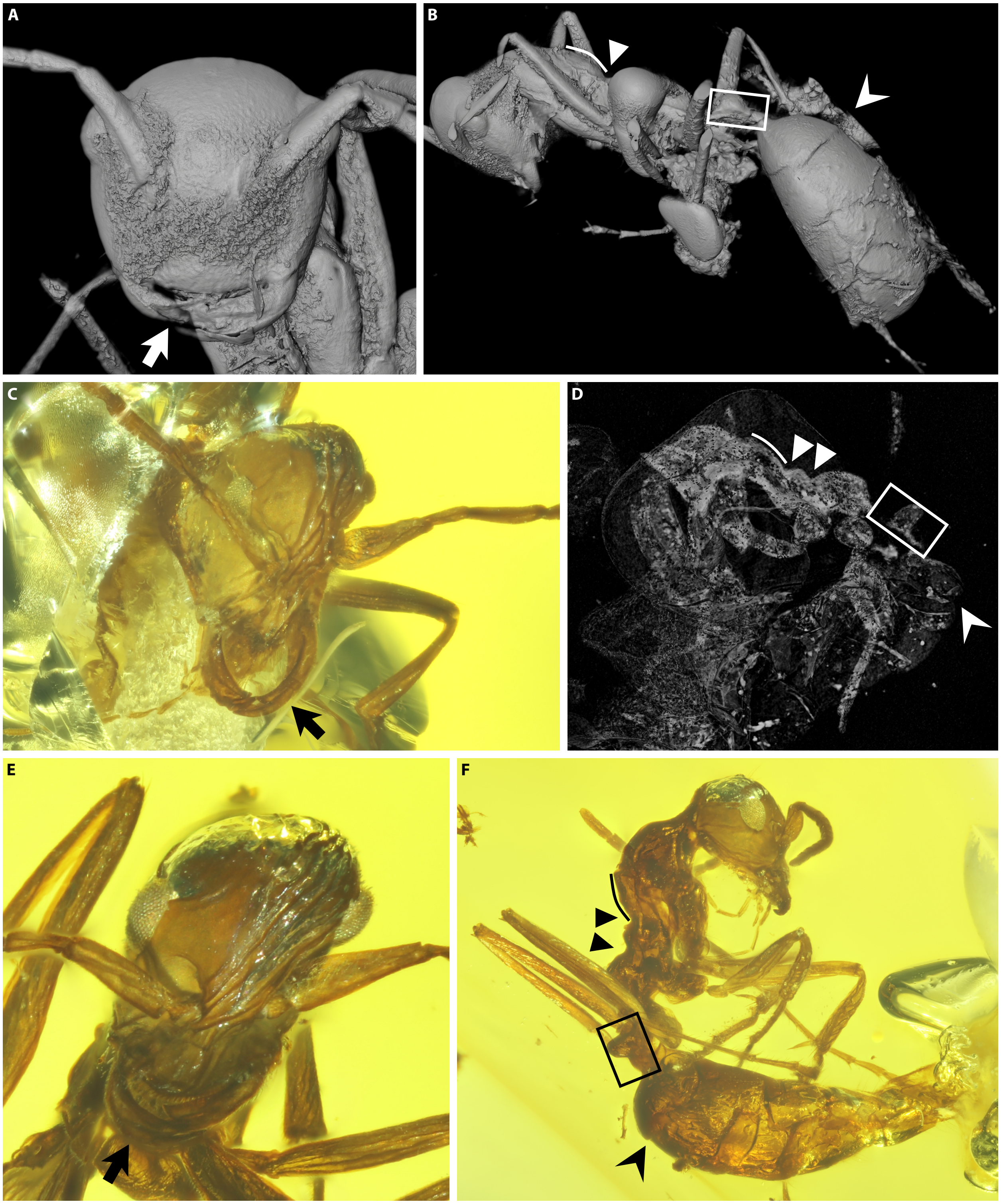
A
B
C
D
E
F

Fig. S1. Taxonomic characters of fossil specimens observed via macrophotography and X-ray CT.

(**A** and **B**) AMNH JZC Bu109. (**C** and **D**) Specimen A in AMNH Bu-KL B1-21 (**E** and **F**) Specimen B in AMNH Bu-KL B1-21. Bidentate mandibles fitting to each clypeus and clypei with setae and without lobate process are indicated by arrows in (A), (C), and (E). Smooth mesonota without apparent ridges are indicated by lines, not constricted fourth abdominal segments are indicated by arrowheads, petioles longer than tall are indicated by framed boxes, and broad mesometanotal and metanotopropodeal sulci are indicated by triangles in (B), (D), and (F). In JZC Bu109 (B), the metanotopropodeal sulcus is hidden by a debris inclusion.

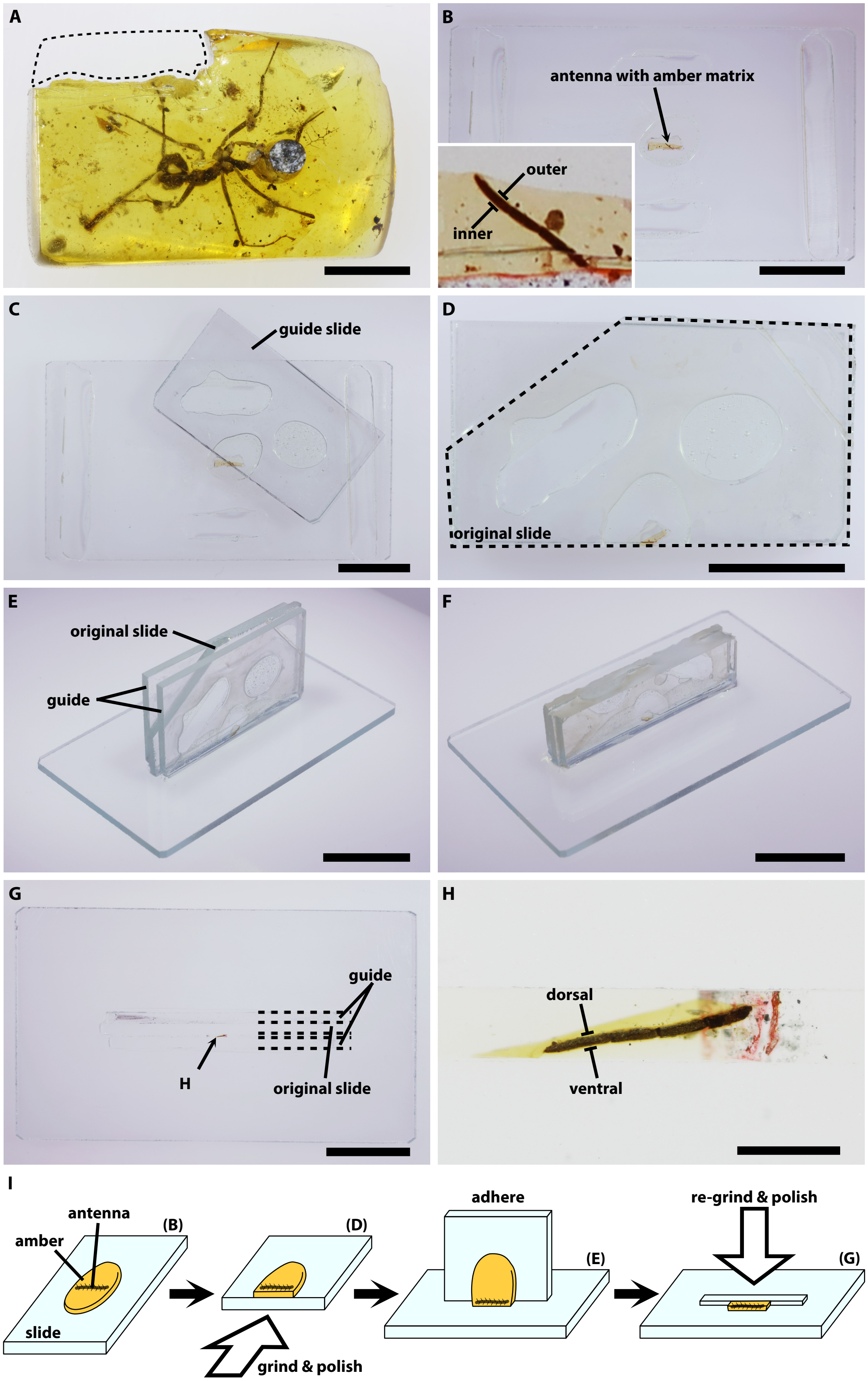

A
B
antenna with amber matrix
outer
inner
C
guide slide
D
original slide
E
original slide
guide
F
G
guide
H
original slide
H
dorsal
ventral
I
antenna
amber
(B)
slide
(D)
grind & polish
adhere
(E)
re-grind & polish
(G)

Fig. S2. Photographic guide and schematic illustration of rotation imaging of amber microinclusions.

(**A**) A fossilized ant in an amber piece (ventral view). The amber piece with the left antenna was cut off and thin-sectioned via a method developed by Taniguchi et al. (*30*) (surrounded area with a dotted line). (**B**) Thinned amber containing the antenna on the slide glass and a higher magnification image of the center area. (**C**) Specimen sandwiched between glass slides for guide. The guide slides were adhered along the antenna with an epoxy resin adhesive, indicating the vertical plane. (**D**) Specimen with the guide slides (cut, ground, and polished from [C]). The original slide is shown by a dotted line. (**E**) Specimen adhered orthogonally on a new slide. (**F**) Specimen ground in approximately half. (**G**) Completely re-thinned specimen. (**H**) Higher magnification image of the center area of (G). The antenna was rotated 90 degrees from the initial thin section in (B). (**I**) Simplified overall procedure for the re-thin sectioning. Each step corresponds to (B), (D), (E), and (G), respectively. Scale bars: 2 mm in (A); 10 mm in (B) to (G); and 0.5 mm in (H).

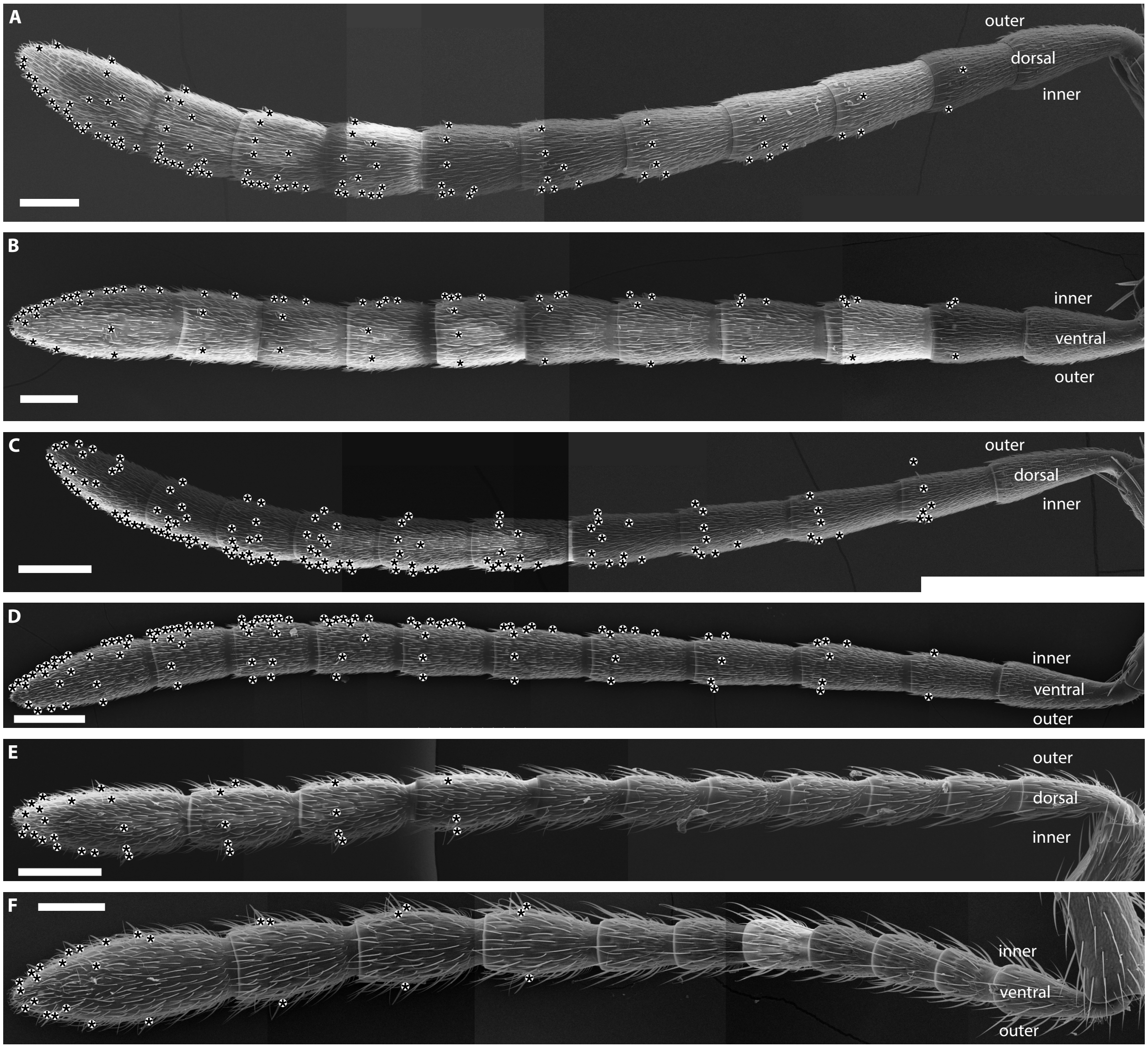
A
outer
dorsal
inner
B
inner
ventral
outer
C
outer
dorsal
inner
D
inner
ventral
outer
E
outer
dorsal
inner
F
inner
ventral
outer

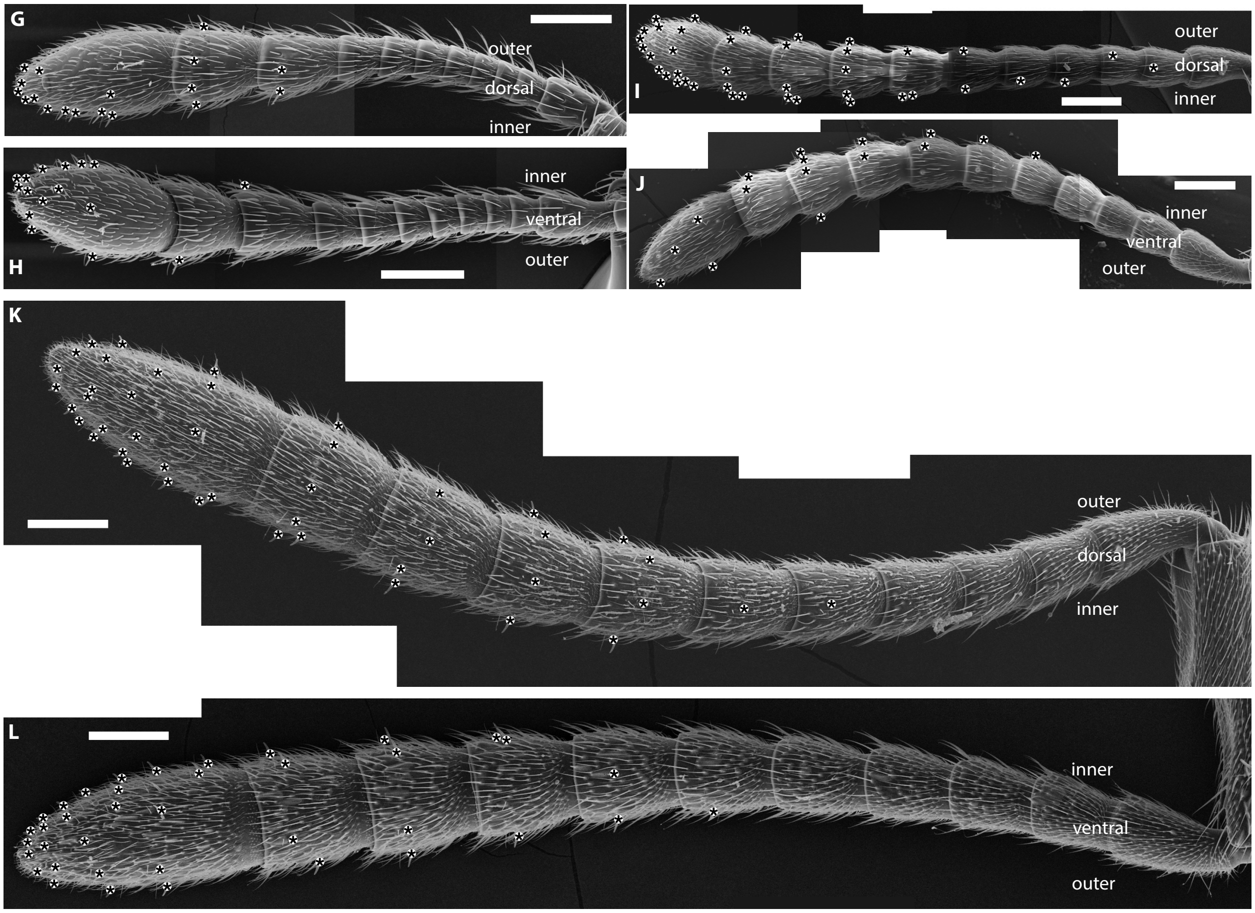
G
outer
dorsal
inner
I
outer
dorsal
inner
H
inner
ventral
outer
J
inner
ventral
outer
K
outer
dorsal
inner
L
inner
ventral
outer

Fig. S3. Distribution pattern of sensilla basiconica of extant ant species.
(**A** and **B**) *Camponotus quadrinotatus*. (**C** and **D**) *Formica sanguinea*. (**E** and **F**) *Aphaenogaster famelica*. (**G** and **H**) *Tetramorium tsushimae*. (**I** and **J**) *Dolichoderus sibiricus*. (**K** and **L**) *Pachycondyla chinensis*. Sensilla basiconica marked by asterisks are biased on the dorsal and inner surfaces on the antennae. Scale bars: 100 µm in (A), (B), and (F) to (L), and 200 µm in (C) to (E).
